# Protective Role of *Spirulina platensis* against Acute Deltamethrin-Induced Toxicity in Rats

**DOI:** 10.1371/journal.pone.0072991

**Published:** 2013-09-09

**Authors:** Mohamed M. Abdel-Daim, Said M. M. Abuzead, Safaa M. Halawa

**Affiliations:** 1 Pharmacology Department, Faculty of Veterinary Medicine, Suez Canal University, Ismailia, Egypt; 2 Physiology Department, Faculty of Veterinary Medicine, Suez Canal University, Ismailia, Egypt; 3 Department of Plant Protection, Faculty of Agriculture, Benha univeristy, Benha, Egypt; National Institutes of Health, United States of America

## Abstract

Deltamethrin is a broad-spectrum synthetic pyrethroid insecticide and acaricide widely used for agricultural and veterinary purposes. However, its human and animal exposure leads to hepatonephrotoxicity. Therefore, the present study was undertaken to examine the hepatonephroprotective and antioxidant potential of *Spirulina platensis* against deltamethrin toxicity in male Wistar albino rats. Deltamethrin treated animals revealed a significant increase in serum biochemical parameters as well as hepatic and renal lipid peroxidation but caused an inhibition in antioxidant biomarkers. Spirulina normalized the elevated serum levels of AST, ALT, APL, uric acid, urea and creatinine. Furthermore, it reduced deltamethrin-induced lipid peroxidation and oxidative stress in a dose dependent manner. Therefore, it could be concluded that spirulina administration able to minimize the toxic effects of deltamethrin by its free radical-scavenging and potent antioxidant activity.

## Introduction

Deltamethrin (DLM) is a broad-spectrum synthetic dibromo-pyrethroid insecticide [α-cyano- 3-phenoxybenzyl-(1R, S)-cis, trans-3-(2,2-dibromovinyl) -2,2-dimethylcyclopropanecarboxylate], widely used to protect agricultural crops, vegetables and fruits against pests such as ants, mites, beetles and weevils. It is also used for nurseries, golf courses, urban structural and landscaping sites, residential home and garden pest control. Moreover, it is used topically in farm animals as an ectoparasiticide against ticks, mites, fleas and flies to control vector-borne diseases [1]. Owing to its rapid metabolism and low toxicity to humans and other non-target animals as well as its high potency on a large number of pests, it has become of choice in most countries [2]. DLM represents an environmental and industrial pollutant that is toxic to animals, birds, fishes and human being living in the same ecosystem and directly or indirectly at the risk of exposure leading to substantial hazards [3].

The main mechanism of DLM as acaricidal and insecticidal effects is believed to result from its binding to a distinct receptor site on voltage-gated sodium channels and prolonging the open state by inhibiting channel deactivation and inactivation [4]–[5]. However, due to its high hydrophobicity, DLM could exert other effects on biological membranes at sites other than the voltage-dependent sodium channel [6]–[7]. According to some reports, the liver was found to accumulate a greater concentration of metabolites since it is the major site of DLM metabolism and kidney is main organ of excretion [8]–[10].

A number of studies on the side effects of this insecticide have been reported, including neurotoxicity [11], allergy and immunosupression [12]–[14], cardiovascular [15] and reproductive side effects [16]. In addition, hepatotoxicity and nephrotoxicity [2], [17] has also been induced.

*Spirulina platenesis* (SP) is a unicellular cyanbacterium, with high nutritional value and with wide range of medicinal applications. It contains very potent naturally occurring antioxidant and free radical scavenging agents. Besides the free radical scavenging and antioxidant activity, spirulina and its active constituent; C-phycocyanin exhibit antiinﬂammatory, neuroprotective, hepatoprotective and immunomodulatory and anticancer activities [18]–[20]. Furthermore, spirulina has been reported to ameliorate organ toxicities induced by heavy metals [21]–[25]. Increased interest in spirulina is based on the fact that, they are believed to be non toxic, bioavailable and provide significant multiorgan protection against many drugs and chemicals induced toxic assaults [26]–[29]. Consequently, the aim of the present study was to investigate the hepatorenoprotective and antioxidant effect of SP against DLM-induced oxidative stress and hepatonephrotoxicity in rats.

## Materials and Methods

### Chemicals

Deltamethrin (Butox® 50 mg/ml) was purchased as a commercial product in clinical formulation from (Intervet Co., France). Pure premium *Spirulina platensis* powder was purchased from (HerbaForce, UK). All kits were purchased from Biodiagnostics Co. (Cairo, Egypt) except Lactate dehydrogenase (LDH), which was purchased from Randox Laboratories Ltd, U.K. All other chemicals used in the experiment were of analytical grade.

### Animals and experimental design

Forty male Albino Wistar rats, weighing 175±25 g, were purchased from The Egyptian Organization for Biological Products and Vaccines. Rats were kept in ventilated room under controlled laboratory conditions of normal light –dark cycle (12 h light/dark) and temperature (25±2°C). Food and water were provided ad libitum. Experimental design and animal handling were approved by the Research Ethical Committee of the Faculty of Veterinary Medicine, Suez Canal University, Ismailia, Egypt (the approval no 20145). All efforts were made to minimize animal suffering.

After 1 week acclimation period, rats were randomly assigned to five different groups; 8 animals each. The 1^st^ group administered normal saline and act as a control group. The 2^nd^ group received oral dose of SP at 1000 mg/kg BW. The 3^rd^ group was orally received DLM at a dose of 30 mg /kg BW (1/5 LD_50_). The 4^th^and 5^th^ groups were given SP at doses of 500 and 1000 mg /kg respectively 1 h before DLM administration at the same dose of group 3. All treatments were continued for 5 days.

### Serum collection and tissue preparation

At the end of experimental period (24 hs after the last DLM dose), blood samples were collected via retroorbital bleeding under light ether anaesthesia. Blood samples were left to clot at room temperature, and then centrifuged at 3000 rpm for 15 minutes. Sera were then, separated and stored at −20°C as aliquots for further biochemical analysis.

After blood collection, rats were sacrificed by decapitation. Liver and kidney were rapidly excised from each animal, trimmed of connecting tissue, and washed free of blood with 0.9% NaCl solution and distilled water. They were then blotted over a piece of filter paper. The tissues were perfused with a 50 mM (sodium phosphate buffer saline (100 mM Na2HPO4/NaH2PO4) (PH 7.4) in an ice-containing medium, containing 0.1 mM ethylene di amine tetra acetic acid (EDTA) to remove any red blood cells and clots. Then tissues were homogenized in 5–10 ml cold buffer per gram tissue and Centrifuged at 5000 r.p.m for 30 min. The resulting supernatant was transferred into Eppendorf tubes, and preserved at −80°C in a deep freezer until used for various biochemical assay.

### Serum biochemical analysis

Freshly separated sera were used for estimation of serum liver and renal injury biomarkers according to manufacturer protocol. Serum alanine aminotransferase (ALT) and aspartate aminotransferase (AST) were evaluated according to Reitman and Frankel, 1957 [30], Alkaline phosphatase (ALP) according to Tietz et al., 1983 [31]. Serum LDH activity was determined enzymatically according to Babson and Babson, 1973 [32], using kits from Randox Laboratories Ltd, U.K., according to the manufacturer's protocol. The enzyme activity was expressed as units/liter computed directly from the absorbance values. Cholesterol was measured according to Richmound, 1973 and Allain et al., 1974 [33]–[34]. Serum total protein was measured according to Lowry et al., 1951 [35]. Total bilirubin was determined according to Schmidt and Eisenburg, 1975 [36]. Renal products; creatinine was determined according to Larsen, 1972 [37], urea according to Coulombe and Favreau, 1963 [38] and uric acid according to Whitehead et al.,1991 [39].

### Evaluation of tissue lipid peroxidation and antioxidant enzymes

Hepatic and renal lipid peroxidation content was evaluated by measurement of MDA according to Mihara and Uchiyama, 1978 [40]. Oxidative stress markers were assessed; superoxide dismutase (SOD) according to Nishikimi et al.,1972 [41], catalase (CAT) according to Aebi, 1984 [42], reduced glutathione (GSH) according to Beutler et al. 1963 [43] and total antioxidant capacity (TAC) according to Koracevic et al., 2001 [44].

### Histopathological examination

Liver and kidney sections were taken immediately from the liver and kidney, fixed in 10% buffered formalin, dehydrated in ethanol (50–100 %), cleared in xylene, and embedded in paraffin. Sections (4–5 μm thick) were prepared and then stained with hematoxilin and eosin (H–E). The sections were examined for the pathological findings of hepatic and renal changes.

### Statistical analysis

All data were expressed as means ± S.E.M. and statistically analyzed using SPSS (Statical Pucteage for Social Science) version 16.0 for Windows (SPSS Inc, Chicago, IL). Statistical significance of differences among different study groups was evaluated by one-way analysis of variance (ANOVA) (P≤0.05). Duncan's multiple range test was used to differentiate between means (to determine differences between means of treatments at significance rates of 0.05).

## Results

### Serum biochemical analysis

The effects of DLM intoxication as well as the preventive effects of SP on serum biochemical analysis are shown in table 1. Significant increases (P≤0.05) in serum liver function marker enzymes (AST, ALT, ALP and LDH) were recorded in DLM intoxicated rats as compared to untreated control group (144.38%, 287.1%, 149.8% and 168.16%, respectively). Similarly, a significant increase (P≤0.05) in serum cholesterol and total bilirubin levels was observed (136.25% and 127.52% respectively). Serum total protein was decreased (77.42%) significantly (P≤0.05) in DLM intoxicated rats as compared to the untreated control group. Renal products; uric acid, urea and creatinine were significantly elevated (295.72%, 253.34% and 1219.18%, respectively) compared to the non-toxicated non-treated group.

**Table 1:** Serum enzymes activity and biochemical parameters in control and different treated groups.

			Experimental groups		
Parameters	Control	SP1000	DLM control	DLM-SP500	DLM-SP1000
AST (u/l)	22.76^a^±0.97	21.38^a^±0.98	32.86^b^±1.69	26.70^c^±1.13	24.44^c^±0.99
ALT (u/l)	28.46^a^±1.48	27.67^a^±1.38	81.71^b^±4.87	55.03^c^±1.18	44.57^d^±1.52
ALP (u/l)	71.86^a^±2.76	68.30^a^±2.26	107.13^b^±2.74	95.50^c^±1.32	85.38^d^±2.17
LDH (u/l)	41.52^a^±2.28	39.39^a^ ±1.07	69.82^b^±2.70	52.80^c^±1.68	44.92^a^±1.89
Cholesterol (mg/dl)	61.30^a^±1.02	59.37^a^±1.12	83.52^b^±1.06	71.51^c^±1.39	66.22^d^±2.05
T.protein (mg/dl)	8.15^a^±0.11	8.41^a^±0.19	6.31^b^±0.12	7.21^c^±0.09	7.66^d^±0.14
T bilirubin (mg/dl)	1.49^a^±0.02	1.39^b^±0.03	1.90^c^±0.03	1.48^a^±0.03	1.41^a^±0.03
Uric acid (mg/dl)	24.77^a^±0.95	25.24^a^±0.79	73.25^b^±4.22	35.84^c^±1.88	28.06^a^±1.80
Urea (mg/dl)	26.53^a^±1.34	25.82^a^±1.51	67.21^b^±2.43	47.01^c^±1.71	41.10^d^±1.66
Cretinine (mg/dl)	0.73^a^±0.05	0.75^a^±0.04	8.90^b^±0.62	5.39^c^±0.39	2.83^d^±0.28

Data are expressed as means ± SE (n = 8).

Deltamethrin (DLM), Spirulina platensis (SP), aspartate aminotransferase (AST), alanine aminotransferase (ALT), alkaline phosphatase (ALP), lactatic dehydrogenase (LDH), total protein (T protein) and total bilirubin (T bilirubin).

Values having different superscripts within same raw are significantly different (P≤0.05).

Pre-treatment with SP at doses of 500 and 1000 mg/kg, one hour prior to DLM intoxication, ameliorate the changes in most of the studied serum parameters in dose dependent manner. The results indicate that SP effectively reduced DLM-induced hepatorenal toxicity.

Spirulina preadministration at a dose of 500 mg/kg significantly (P≤0.05) reduced the serum hepatic biomarkers; AST, ALT, ALP, LDH, cholesterol and total bilirubin ( about 81.25%, 67.35%, 89.14%, 75.62%, 85,62% and 77.89%, respectively) while, serum total protein was increased ( about 114.26%) in comparison with DLM-intoxicated group ( P≤0.05).There were significant decreases (P≤0.05) in serum renal products; uric acid, urea and creatinine (49.93%, 69.94% and 60.56%, respectively) compared to the DLM-toxicated non-treated group.

Spirulina at a dose of 1000 mg/kg significantly (P≤0.05) reduced the serum hepatic biomarkers; AST, ALT, ALP, LDH, cholesterol and total bilirubin (about 74.38%, 54.55%, 79.7%, 64.34%, 79.29% and 74.21%, respectively). Serum total protein, was increased (about 121.39%) in comparison with DLM-intoxicated group (P≤0.05). The renal products; uric acid, urea and creatinine were significantly reduced (38.31%, 61.15% and 30.8%, respectively) compared to the DLM-toxicated non-treated group.

There were no significant changes in serum biomarkers in rats received SP only at a dose of 1000 mg/kg (2^nd^ group) if compared to the non-toxicated non-treated group, indicating the safety of SP at the selected doses used in this study.

### Hepatic lipid peroxidation and antioxidant status

The effects of DLM intoxication as well as preventive effects of SP on liver tissue homogenate lipid peroxidation and antioxidant parameters are shown in table 2. A significant increase (P≤0.05) in liver MDA content (219.68%) was observed compared with the control group. On the other hand, liver GSH, SOD, CAT and TAC were significantly (P≤0.05) decreased (66.98%, 39.56%, 33.33% and 70.88%, respectively). Concerning DLM-SP500 group, liver MDA was decreased (74.24%) while GSH, SOD, CAT, and TAC were increased (117.38%, 176.53%, 173.33%, and 125.63%, respectively) compared to DLM-intoxicated group. Regarding to DLM-SP1000 group, liver MDA was decreased (65.03%), while GHS, SOD, CAT, and TAC were increased (about 134.68%, 258.3%, 215.56% and 137.27%, respectively).

**Table 2:** Liver oxidative stress marker and antioxidant parameters in control and different treated groups.

	Experimental groups				
Parameters	Control	SP1000	DLM control	DLM-SP500	DLM-SP1000
MDA (nmol/g)	32.52^a^±1.05	28.68^a^±1.38	71.44^d^±2.13	53.04^c^±2.00	46.46^b^±1.81
GSH (mg/g)	54.46^a^±2.00	58.19^a^±1.49	36.48^d^±2.04	42.82^c^±1.46	49.13^b^±1.62
SOD (u/g)	16.91^a^±0.90	18.66^a^±0.70	6.69^c^±0.47	11.81^b^±0.87	17.28^a^±0.72
CAT (u/g)	1.35^a^±0.07	1.50^b^±0.07	0.45^c^± 0.01	0.78^d^± 0.05	0.97^e^± 0.04
TAC (µmol/g)	47.39^a^±1.34	51.80^b^±1.31	33.59^c^±0.68	42.20^d^±1.30	46.11^a^±0.56

Data are expressed as means ± SE (n = 8).

Deltamethrin (DLM), Spirulina platensis (SP), malondialdehyde (MDA), reduced glutathione (GSH), superoxide dismutase (SOD), catalse (CAT), total antioxidant capacity (TAC).

Values having different superscripts within same raw are significantly different (P≤0.05).

### Renal lipid peroxidation and antioxidant status

The effects of DLM intoxication as well as preventive effects of SP on renal tissue homogenate lipid peroxidation and antioxidant parameters are shown in table 3. A significant increase (P≤0.05) in renal MDA content (650.86%) and decreases (P≤0.05) in renal GSH, SOD, CAT and TAC were recorded (67.63%, 83.23%, 86.96%, 64% and 79.1%, respectively) compared with the control group.

**Table 3:** Renal oxidative stress marker and antioxidant parameters in control and different treated groups.

	Experimental groups				
Parameters	Control	SP1000	DLM control	DLM-SP500	DLM-SP1000
MDA (nmol/g)	3.50^a^±0.15	3.03^a^±0.08	22.78^d^±1.26	15.12^c^±0.81	11.67^b^±0.67
GSH (mmol/g)	6.24^a^±0.17	7.23^b^±0.24	4.22^c^±0.32	4.61^c^±0.21	5.65^a^±0.41
SOD (u/g)	16.94^a^±0.93	20.39^b^±0.85	14.10^c^±0.59	16.49^a^±0.43	17.98^a^±0.66
CAT (u/g)	0.25^a^±0.02	0.28^a^±0.02	0.16^b^±0.01	0.20^a^±0.02	0.23^a^±0.02
TAC (µmol/g)	1.34^a^±0.05	1.86^b^±0.04	1.06^c^±0.04	1.38^a^±0.08	1.56^d^±0.07

Data are expressed as means ± SE (n = 8).

Deltamethrin (DLM), Spirulinaplatensis (SP), malondialdehyde (MDA), reduced glutathione (GSH), superoxide dismutase (SOD), catalse (CAT), total antioxidant capacity (TAC).

Values having different superscripts within same raw are significantly different (P≤0.05).

Concerning DLM-SP500 group, renal MDA decreased (66.37%) significantly (P≤0.05), while SOD, CAT and TAC were increased (116.95%, 125% and 130.19%, respectively) compared to DLM-intoxicated group. There was a non significant decrease observed in GSH compared with DLM-intoxicated group. Regarding to DLM-SP1000 group, renal MDA (51.23%) was decreased. On the other hand, GHS, SOD, CAT and TAC were increased (about 133.89%, 127.52%, 143.75% and 147.17%, respectively).

### Histopathological study

**Figure 1:**
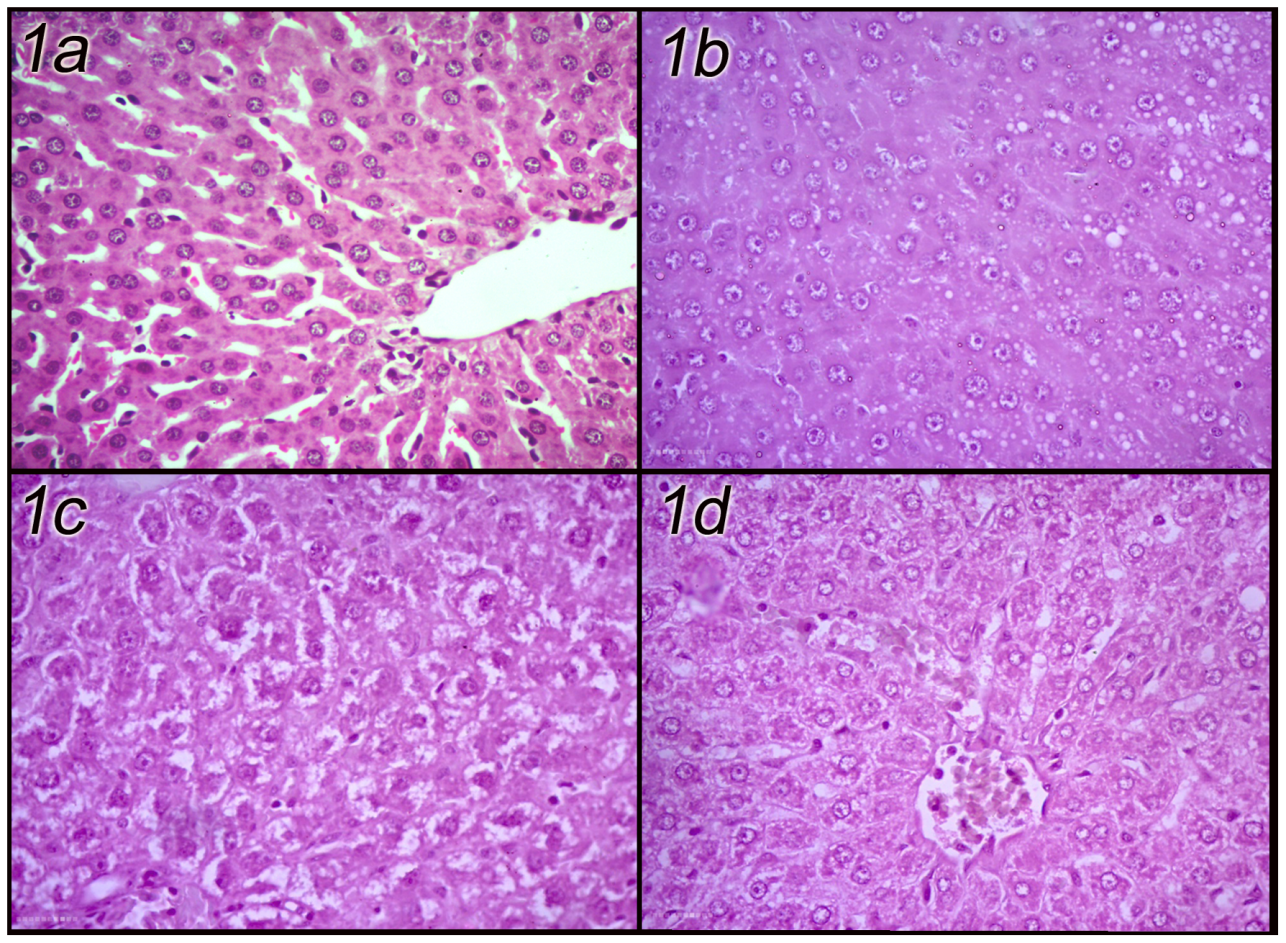
Hepatic sections of control, DLM-intoxicated and DLM-SP treated rats. (H&E X400). (a: Control, b: DLM-intoxicated, c: DLM-SP500, d: DLM-SP1000).

The effects of DLM intoxication as well as the protective effects of SP were assessed histopathologically in hepatic and renal sections. The liver of DLM administered rats showed disrupted hepatic architecture and the hepatocytes were dissociated from each others. Multifocal hepatocytes showed multiple clear rounded vacuoles within the cytoplasm (vacuolar degeneration). Occasionally some hepatocytes were shrunken, hyperesinophilic and dissociating from neighboring cells (apoptotic cells). The portal area expanded with few inflammatory cells; lymphocytes and plasma cells. Preadministration of SP enhanced the hepatic picture in a dose dependent manner. In DLM-SP500 group, the rat liver showed a slight loss of hepatic architecture especially around the central vein, and the hepatocyte cytoplasmic vacuolation is milder than that of the control intoxicated group. In DLM-SP1000 the liver retained its architecture and the hepatocytes had indistinct borders with no necrosis noticed (Fig. 1).

**Figure 2:**
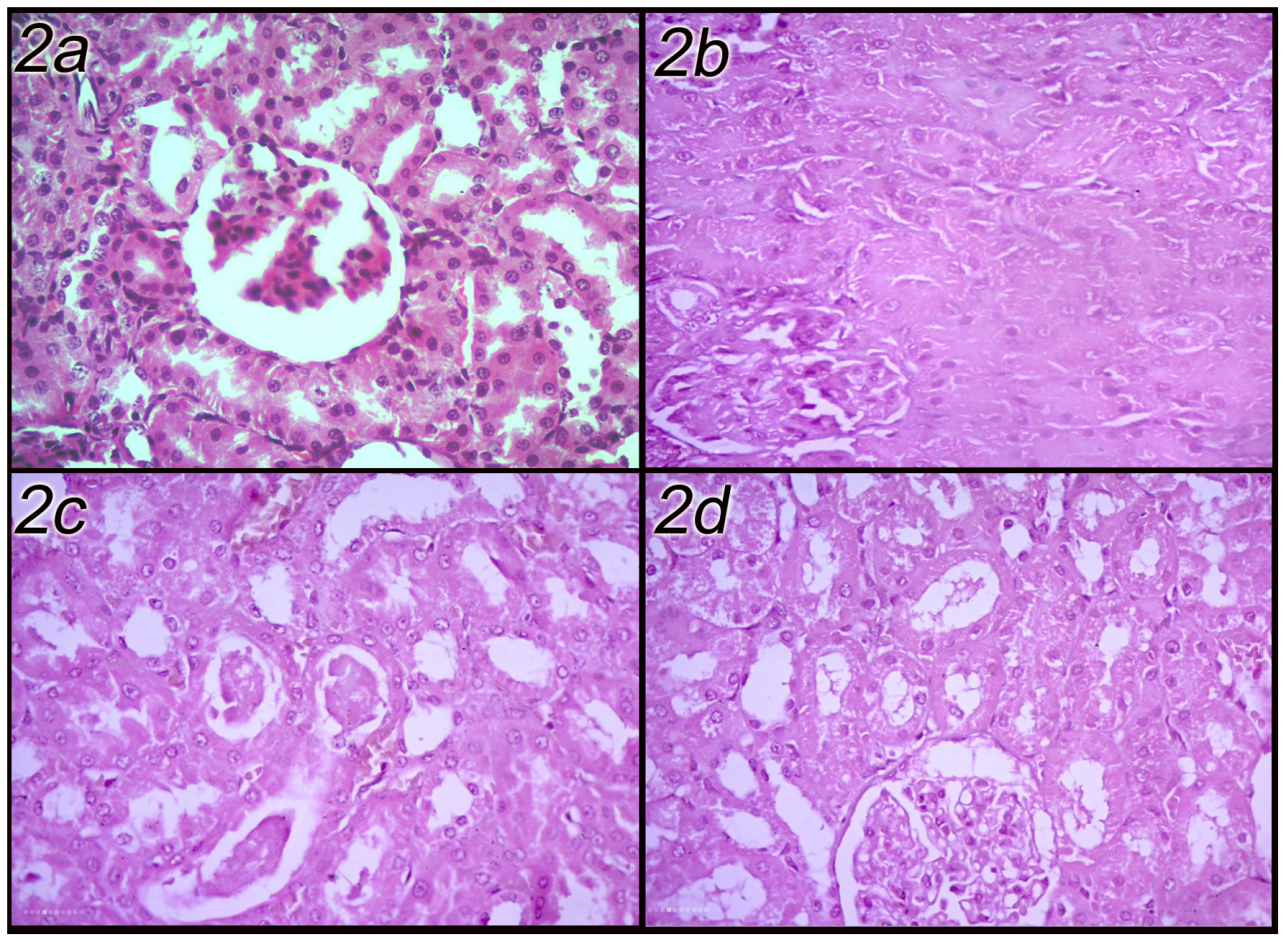
Renal sections of control, DLM-intoxicated and DLM-SP treated rats. (H&E X400). (a: Control, b: DLM-intoxicated, c: DLM-SP500, d: DLM-SP1000).

The kidney of DLM administered rats showed a cortex with tubular cellular swelling leading to obliteration of the tubular lumen. Few tubules were lined with attenuated epithelium. Other tubules had narrow, star-shaped lumen. The tubular cells had a granular eosinophilic cytoplasm indicating acute tubular injury. Preadministration of SP enhanced the renal picture in a dose dependent manner. In DLM-SP500 group, the renal tubules had many casts and desquamated epithelium within the lumen, while rats in DLM-SP1000 group had almost normal renal tubules, containing little casts with congestion of interlobular capillaries (Fig. 2).

## Discussion

Reactive oxygen species (ROS) are continuously generated inside the mammalian body as a consequence of exposure to a plenty of exogenous drugs and xenobiotics in our environment and/or a number of endogenous metabolic processes involving redox enzymes and bioenergetic electron transport mechanism [45]. Under normal circumstances, the ROS generated are neutralized by the endogenous antioxidants and there is an equilibrium between the ROS generated and the antioxidants present [45]. Deleterious effects caused by ROS occur as a consequence of an imbalance between the formation and inactivation of these species leading to irregularities in cellular physiology and different pathological conditions [45].

The ROS readily induce oxidative damage to various biomolecules including DNA, lipids, proteins, and lipoproteins [46]. This oxidative damage has been considered as an important etiological factor implicated in several chronic diseases such as neurodegenerative diseases, diabetes mellitus, atherosclerosis, arthritis, and cancer [47]. Oxidative stress affects many cellular functions through various mechanisms such as alteration in gene expression by activation of transcription factor NF-kB or induction of mitochondrial permeability with lethal consequences [48].

Deltamethrin; a pyrithroid insecticide has been widely used in industrial agriculture and veterinary practice worldwide that would be potentially an exposure risk to workers in this field [1]. Although several reports about the toxicity of DLM have been published, little study has been performed about the use of natural products for prevention of such toxicity and mechanism of their ameliorative action.

In the present study, hepatorenal injuries caused by DLM may be attributed to the oxidative stress resulted from free radical production. DLM intoxication increased serum liver injury biomarkers; AST, ALT, ALP, LDH, cholesterol and total bilirubin. Moreover, it reduced serum total protein level. Although these biomarkers are not specific for hepatic damage, the increase in their activity reﬂects active liver dysfunction. In addition, it elevated serum renal dysfunction products level such as urea, creatinine and uric acid (Table 1). DLM treatment increased lipid peroxidation through elevated hepatic and renal MDA level, decreased hepatic and renal enzymatic; SOD and CAT as well as non-enzymatic; GSH antioxidant level. (Tables 2&3)

All these effects are involved in the cascade of events leading to DLM-mediated hepatorenal oxidative stress and toxicity. This indicates that hepatorenal injuries induced by DLM is the result of oxidative stress that arise as a result of excessive generation of ROS, which have been reported to attack various biological molecules including lipids and causing lipid peroxidation. The activities of antioxidant enzymes including the enzymes involved in glutathione metabolism were also perturbed in DLM treated group (Tables 2 and 3) indicating the involvement of oxidative stress in DLM-mediated hepatorenal injury. These results are consistent with the literature [2], [16], [49]–[50] and point towards the role of ROS in DLM-mediated injury and toxicity.

Pyrithroid insecticide; DLM caused an increase in AST and ALT enzymes activity as well as MDA levels in rats [2], [51].These alterations might differ dependent on exposure time and dose.

Deltamethrin treatment has led to hepatorenal degeneration in catfish, as indicated by the increased serum levels of AST, ALT, urea and creatinine. At the same time, it increased MDA levels in liver and kidney. Moreover serum total protein and albumin as well as hepatic and renal CAT were reduced [52]. Acute intoxication of deltamethrin in monosex Nile tilapia; *Oreochromis niloticus* led to hepatic damage indicated by the increased serum AST, ALT, ALP and cholesterol levels. In addition, it decreased total protein and albumin levels [53].

In the current study, the pre-administration of SP (500 and 1000 mg/kg) reduced the serum hepatic and renal injury biomarkers. Moreover, it reduced the lipid peroxidation in hepatic and renal tissues. In addition, there were elevations of liver and renal antioxidant enzymes and glutathione levels due to SP administration in a dose dependent manner. The antioxidant and protective effects of SP is owed to their content of antioxidant active constituents such as C-phycocyanins, β carotene, vitamins, minerals, proteins, lipids and carbohydrates reported in SP [54]. Many previous literatures showed the hepatoprotective effects of SP and its active constituents against drugs, chemicals and xenobiotics [24], [55]–[56]. The nephroprotective effects of SP have been reported against renal injury induced by gentamicin [27], [57] as well as oxalate [58]–[59]. Moreover, the antioxidant effects of SP have been reported against sodium fluoride-induced oxidative alterations in offspring of pregnant rats [60]. In another study, SP fed pregnant rats' alleviated lead-induced brain damage in newborns [61].

Pre-treatment with SP might play a role in reducing the toxic effect of cadmium and its antioxidant properties seem to mediate such a protective effect, indicated by the reduction of MDA and NO as well as the elevation of GSH and SOD levels in liver tissue [24]. In another renal injury study, SP at dose of 1000 mg/kg (similar to the dose used in our study) elicited significant renoprotective activity by decreasing MDA, NO, creatinine and urea, while elevating GSH, SOD, GSH-Px level, indicating the therapeutic potential of SP against gentamicin sulphate induced nephrotoxicity and ROS production [57].

The protective effect of SP against DLM-induced oxidative stress in our study could be either direct by inhibiting lipid peroxidation and scavenging free radicals or indirect through the enhancement of the activity superoxide dismutase and CAT; the enzymatic free radicals scavengers in the cells. These properties could be attributed to the high levels of antioxidants such as c-phyocyanin, carotenoids, vitamins, minerals, lipids, proteins and carbohydrates, reported in SP [54]. Therefore, SP could be used to prevent and treat hepatic and renal diseases especially those induced by oxidative damage.

As the liver is the main organ of various metabolic pathways and the kidney is the primary organ of drug and xenobiotic execration, toxic effects of chemicals usually appear primarily in the liver and kidney tissues [62]. The loss of hepatic architecture and hydropic degeneration as well as renal tubular deformities in rats exposed to deltamethrin were observed in the present study indicating acute hepatorenal injury. The histopathological results also confirmed the SP protection against DLM-induced hepatonephrotoxicity.

## Conclusions

Oxidative stress plays a major role in DLM-induced toxicity. Antioxidants have been proven to be effective in ameliorating DLM-induced toxicity in many previous interventions. Spirulina is a potent antioxidant which is reported to to ameliorate the effect of many known chemotherapeutic agents and pesticides as well.

In the present study, it is clear that DLM exposure resulted in varying degrees of lipid peroxidation, inhibition in the antioxidant enzymes activity and alterations of serum biochemical parameters. SP pre-exposure provided near complete protection in terms of serum and tissues biochemical changes, antioxidant enzymes activity and oxidative stress.
